# Supplementary figures and images for: Continental-Scale Microbiome Study Reveals Different Environmental Characteristics Determining Microbial Richness, Composition, and Quantity in Hotel Rooms
Source: mSystems. 2020 May 19;5(3):e00119-20. doi: 10.1128/mSystems.00119-20 (PMC7253364; doi:10.1128/mSystems.00119-20)

A

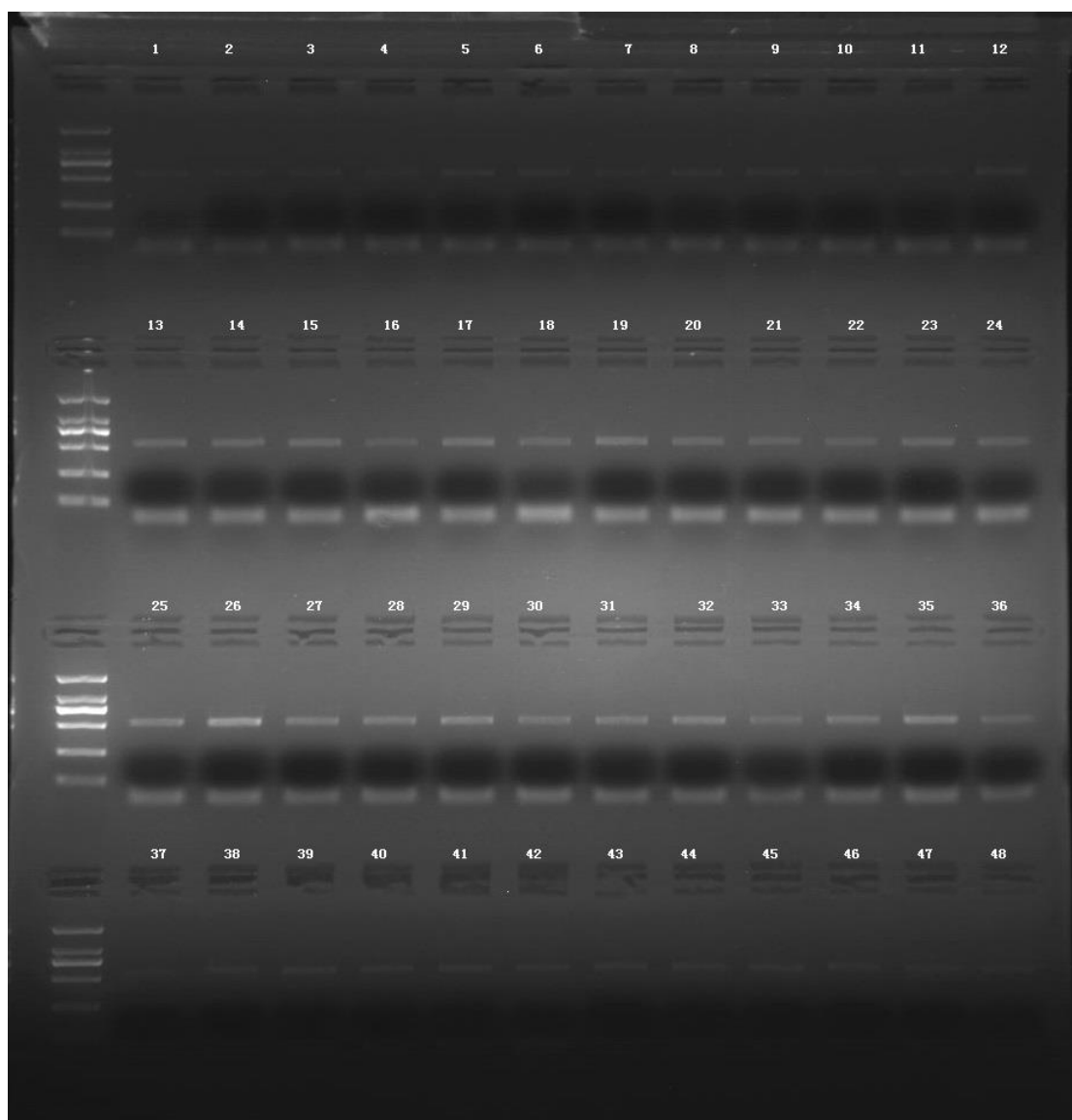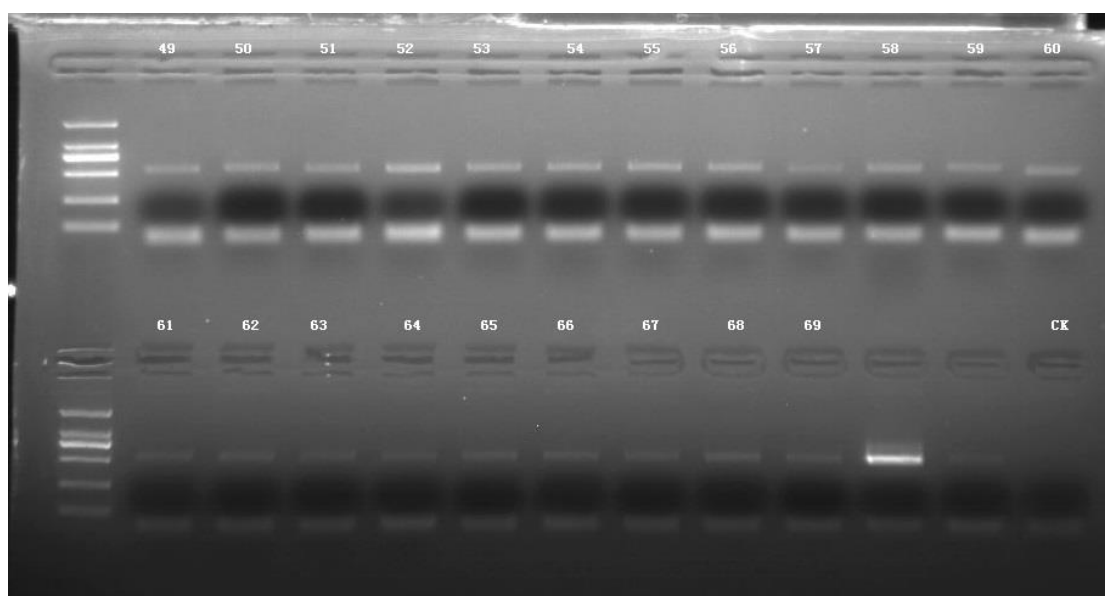

B

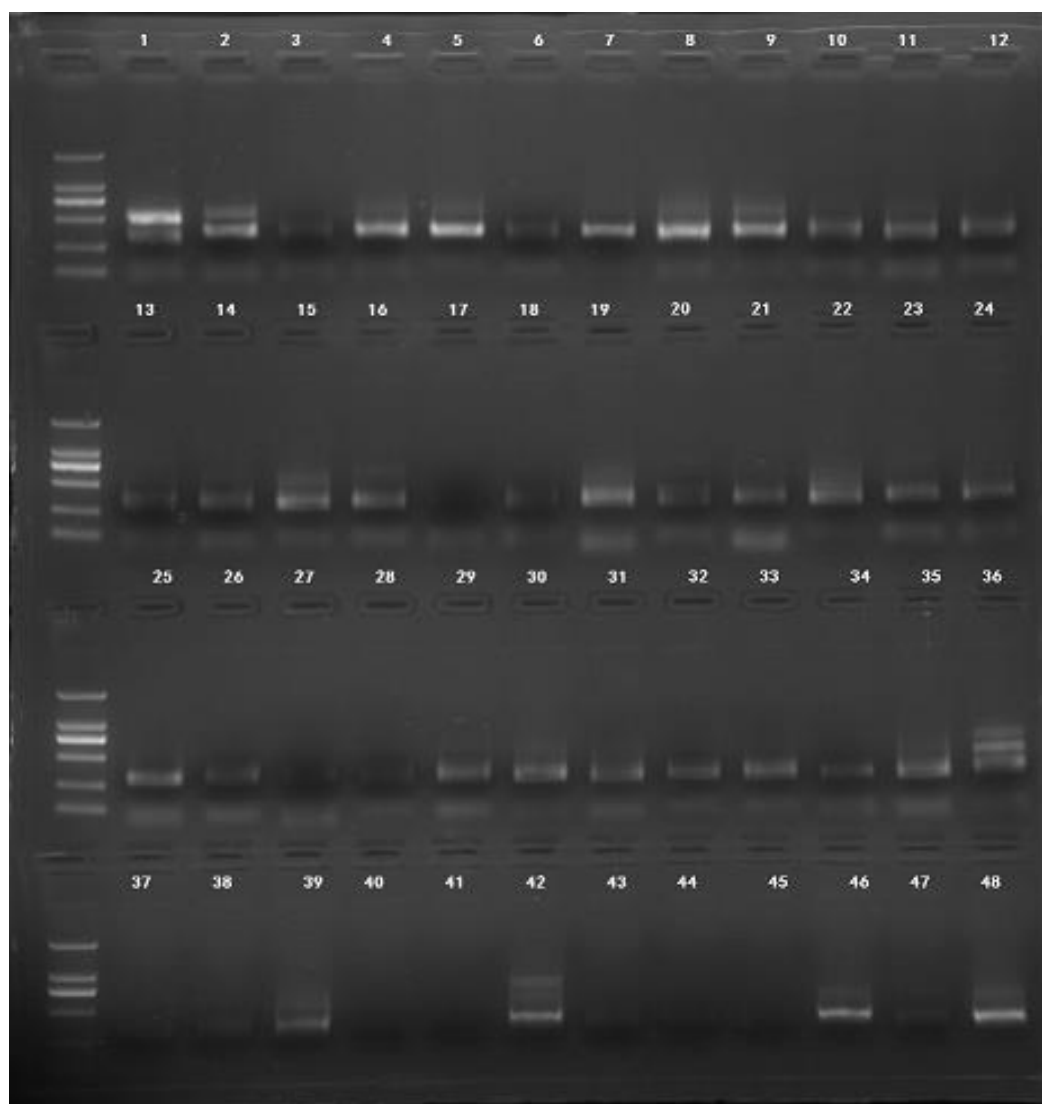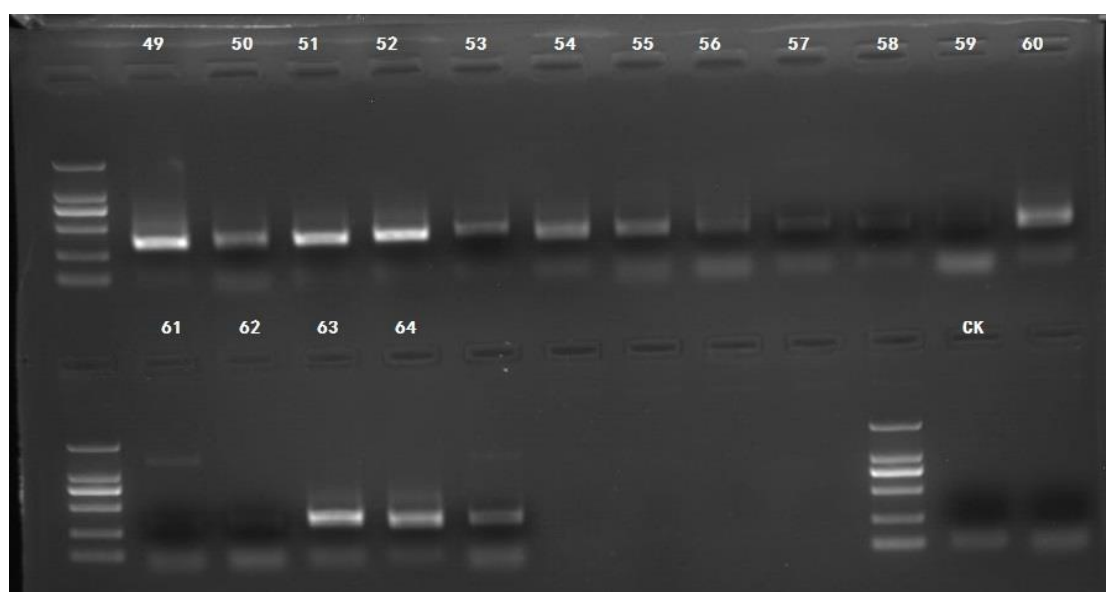

Supplement: FIG S1 [file mSystems.00119-20-sf001.pdf]

A

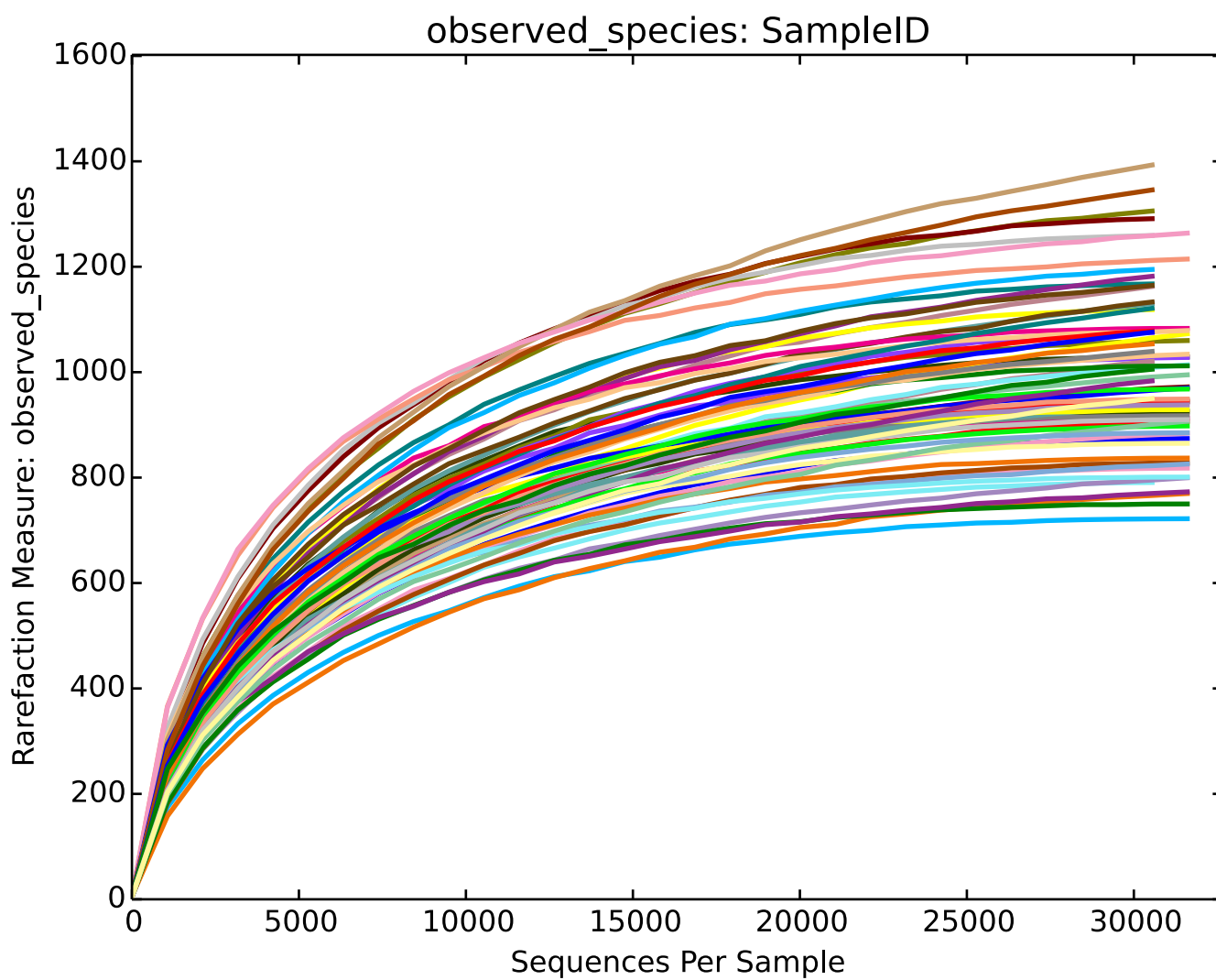

B

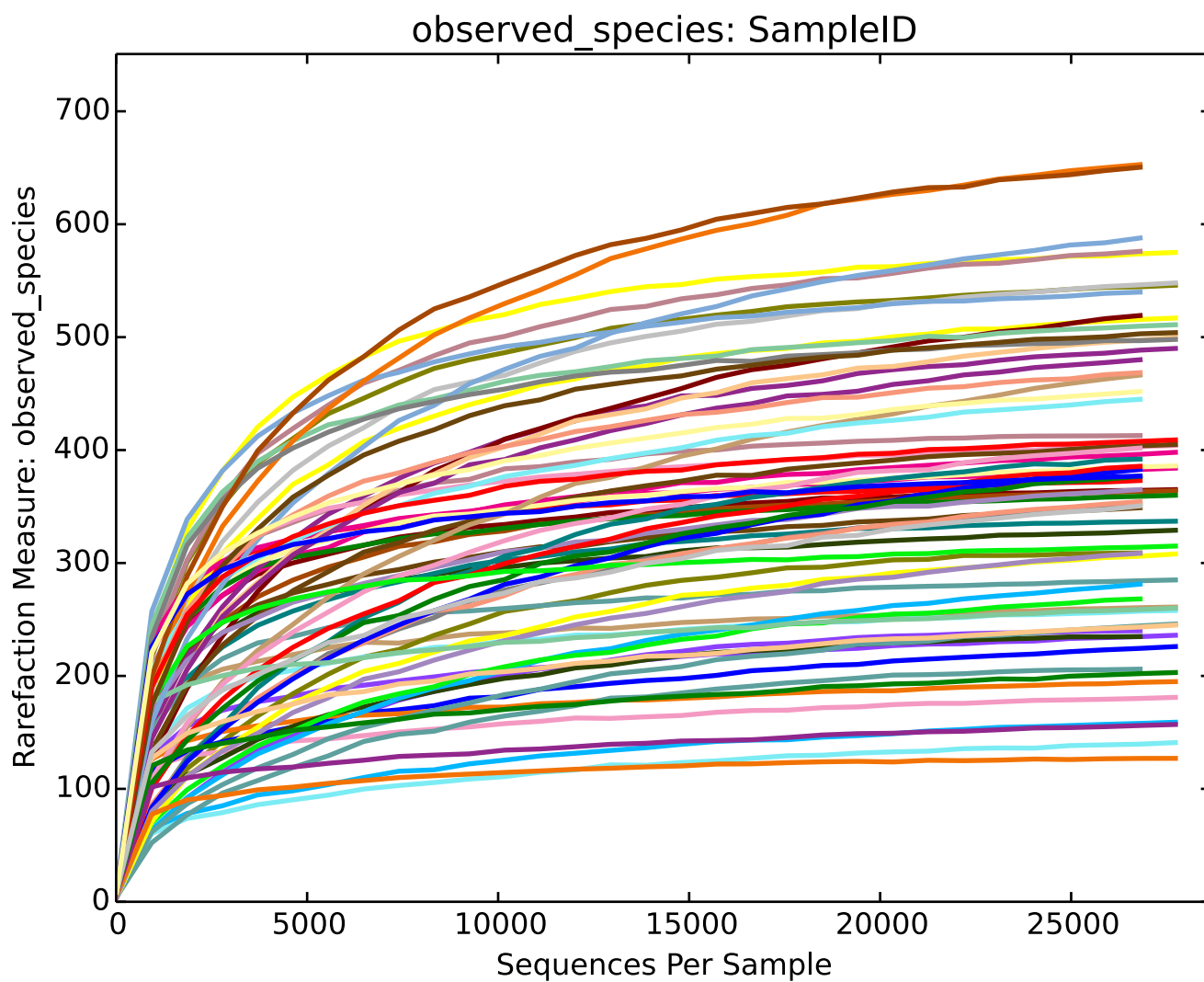

Supplement: FIG S2 [file mSystems.00119-20-sf002.pdf]

A

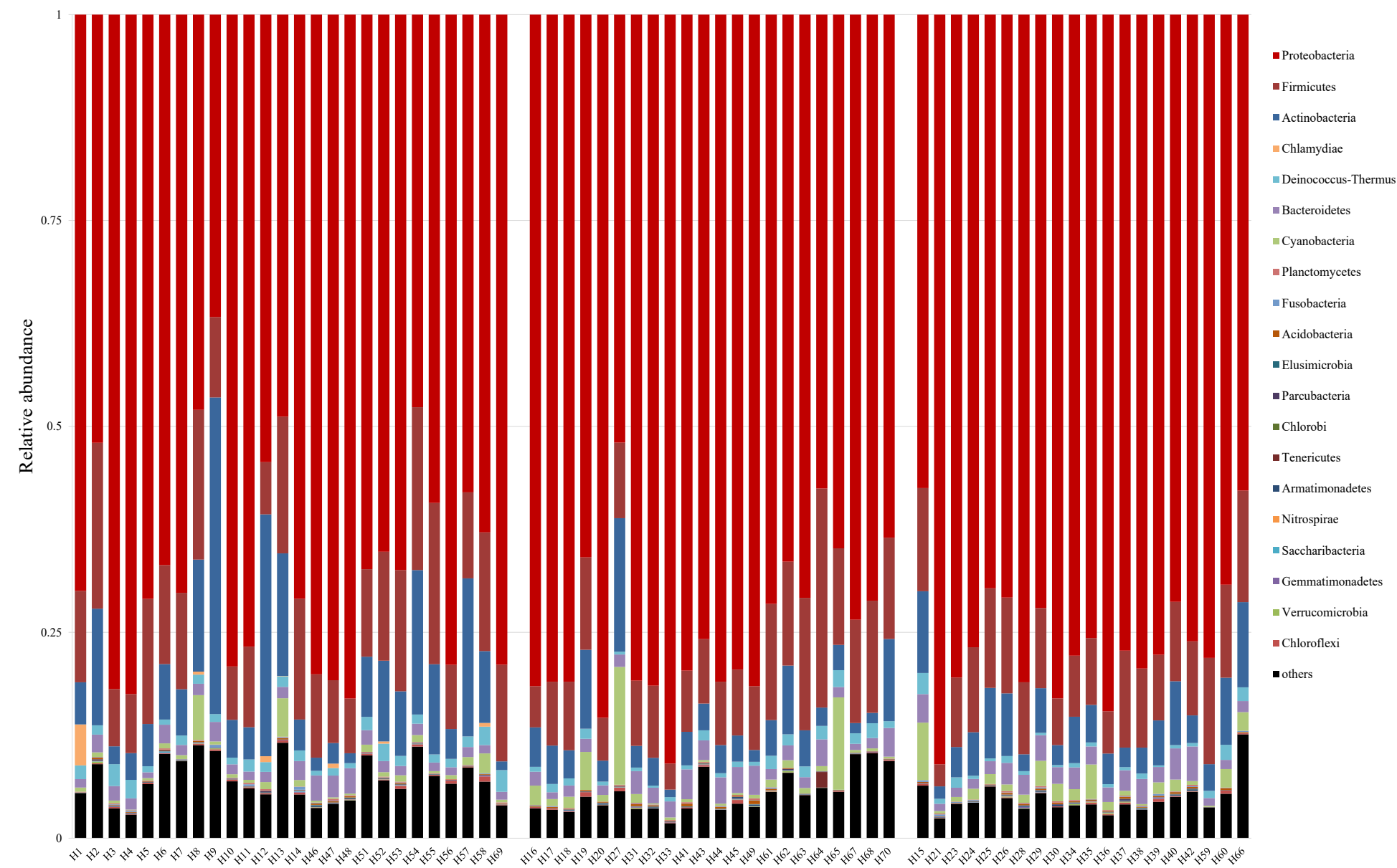

B

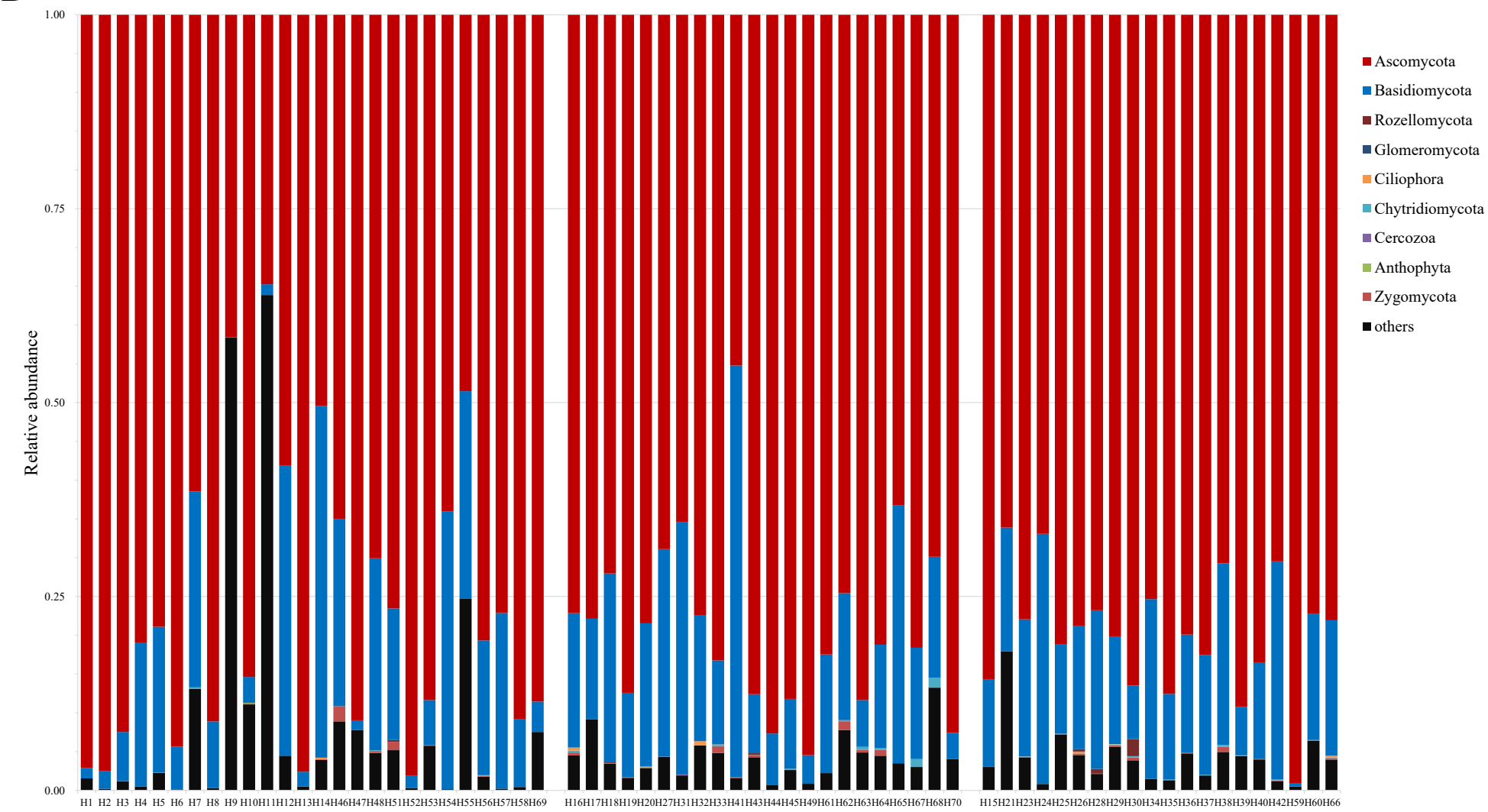

Supplement: FIG S3 [file mSystems.00119-20-sf003.pdf]

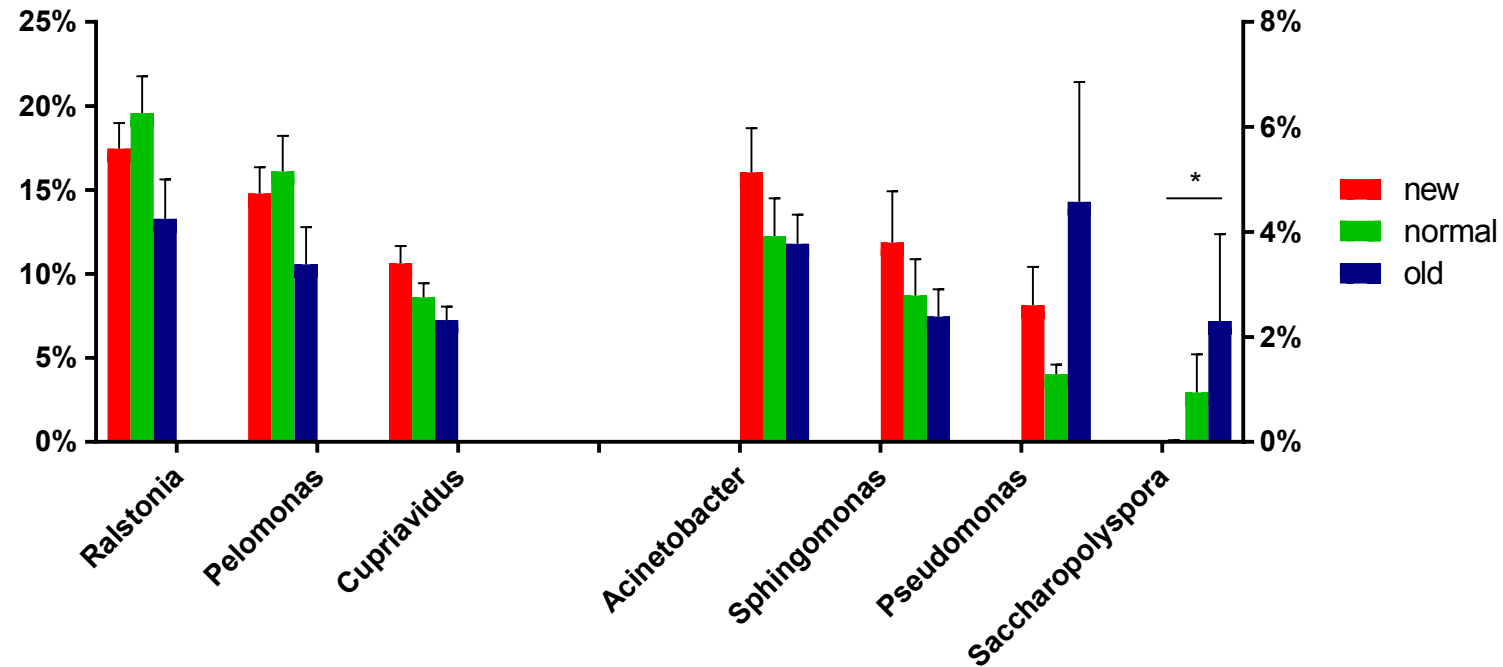

Supplement: FIG S4 [file mSystems.00119-20-sf004.pdf]

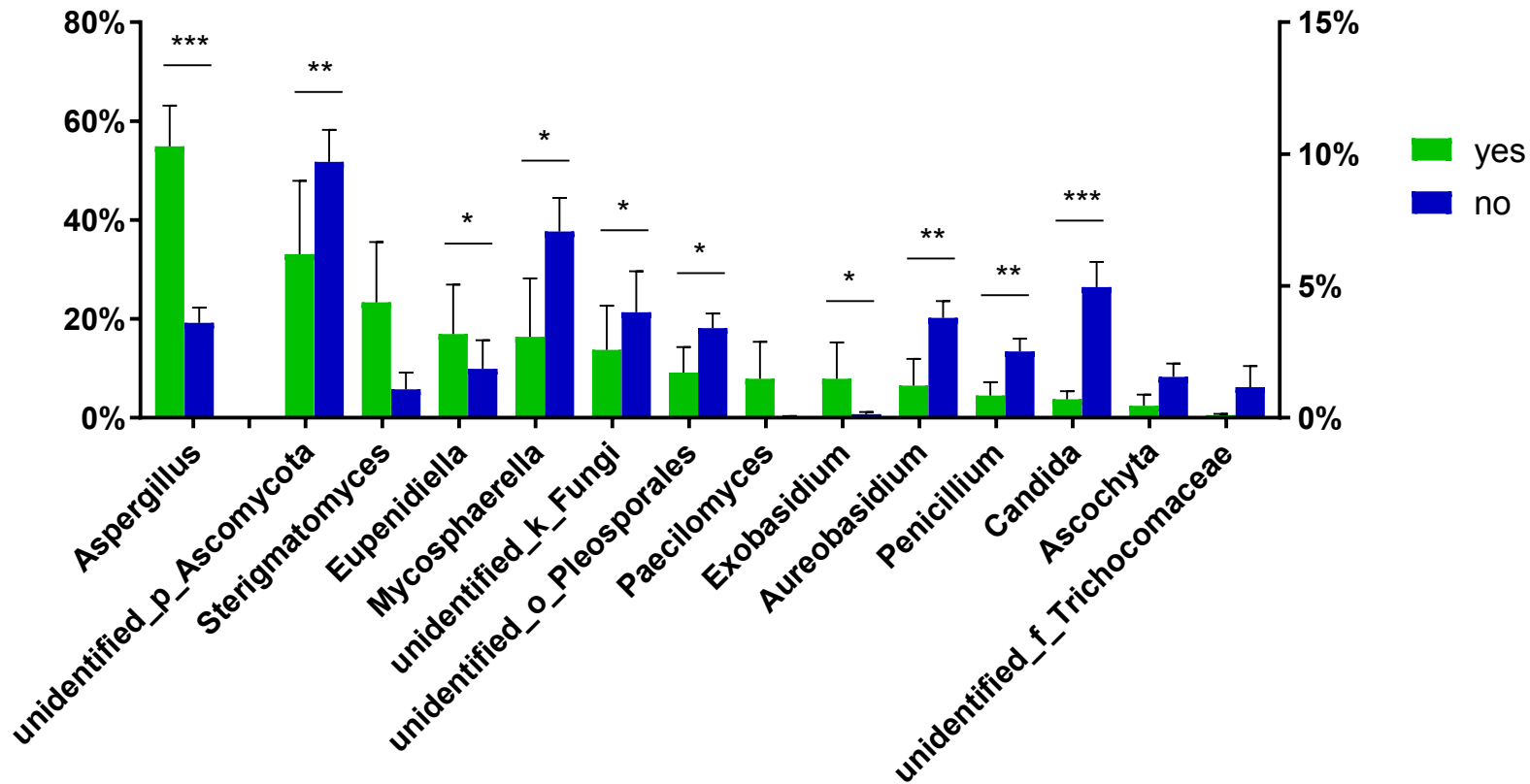

Supplement: FIG S5 [file mSystems.00119-20-sf005.pdf]
